# Open and reusable deep learning for pathology with WSInfer and QuPath

**DOI:** 10.1038/s41698-024-00499-9

**Published:** 2024-01-10

**Authors:** Jakub R. Kaczmarzyk, Alan O’Callaghan, Fiona Inglis, Swarad Gat, Tahsin Kurc, Rajarsi Gupta, Erich Bremer, Peter Bankhead, Joel H. Saltz

**Affiliations:** 1 Department of Biomedical Informatics, Stony Brook University, Stony Brook, NY USA; 2 Centre for Genomic & Experimental Medicine, Institute of Genetics and Cancer, The University of Edinburgh, Edinburgh, UK; 3 Edinburgh Pathology and CRUK Scotland Centre, Institute of Genetics and Cancer, The University of Edinburgh, Edinburgh, UK

**Keywords:** Computational biology and bioinformatics, Cancer imaging, Cancer imaging

## Abstract

Digital pathology has seen a proliferation of deep learning models in recent years, but many models are not readily reusable. To address this challenge, we developed WSInfer: an open-source software ecosystem designed to streamline the sharing and reuse of deep learning models for digital pathology. The increased access to trained models can augment research on the diagnostic, prognostic, and predictive capabilities of digital pathology.

Histopathology is the bedrock of cancer diagnosis and traditionally relies on the examination of physical slides containing human tissue specimens using high-power microscopy. In recent years, the field has been moving towards digital pathology, whereby glass slides are scanned as high-resolution images, known as whole slide images (WSIs). Each individual WSI is typically very large, often over 40 gigabytes uncompressed. The widespread adoption of digital pathology therefore poses considerable challenges for data storage and visualization, but also unlocks the potential to apply computational methods for diagnostics and prognostics.

It is difficult to overstate the transformative effect deep learning has had on digital pathology research. Many studies have suggested the potential for deep learning-based artificial intelligence (AI) methods to revolutionize different aspects of pathology practice, such as by identifying lymphocytic infiltrates, microsatellite instability, genomic aberrations, and other clinically important phenotypes^1–3^. However, the myriad algorithms published in the literature belies a dearth of implementations that are actually usable within the research community. In most cases, it is simply not possible for other research groups to validate the use of published methods on their own images and cohorts. One reason for this is that required data are not available: a recent survey of 161 peer-reviewed studies using deep learning for pathology found that while 1 in 4 shared code, only 1 in 8 shared trained model weights^4,5^. Furthermore, in the minority of cases where code and models are available, they are typically not in a form amenable to pathologists without coding experience to use and explore. The result is that reported findings cannot properly be reproduced and interrogated by the wider community, and the key domain experts — pathologists — often find themselves to be particularly excluded. Tackling problems such as model generalization and overcoming batch effects urgently requires an increase in openness, replicability, and reusability.

In the present paper, we respond to the call to “make deep learning algorithms in computational pathology more reproducible and reusable”^4^ by introducing WSInfer (Whole Slide Inference): a collection of software tools designed to streamline the sharing and reuse of trained deep learning models in digital pathology (Fig. 1). In the current implementation, we have focused on the generic task of patch classification, which is widely used across a broad range of pathology applications with deep learning models that facilitate diagnostic, prognostic, or predictive capabilities. The models currently included in the WSInfer Model Zoo include those for classifying lymphocytic regions, tumor tissue, Gleason grades, and other phenotypes (Table 1); we have used some of the models in collaborative projects that study the immune landscape of cancer^6–9^. We provide an example below of how WSInfer may be used to create spatial maps of tumor and tumor-infiltrating lymphocytes (TILs) in WSIs, which have been suggested to be both prognostic and predictive in several cancers^6,10–12^. Because WSIs are so big, they are typically broken into patches to make analysis practicable. Trained patch-based deep neural networks are typically applied across a WSI to classify patches into different tissue components (e.g. tumor, stroma, lymphocytes) or make predictions directly related to patient outcome. The output of patch classification is typically a spatial classification map, which can often be integrated across the WSI to create a single output representing a diagnosis, prediction, or ‘score’ for that slide.

**Fig. 1:**
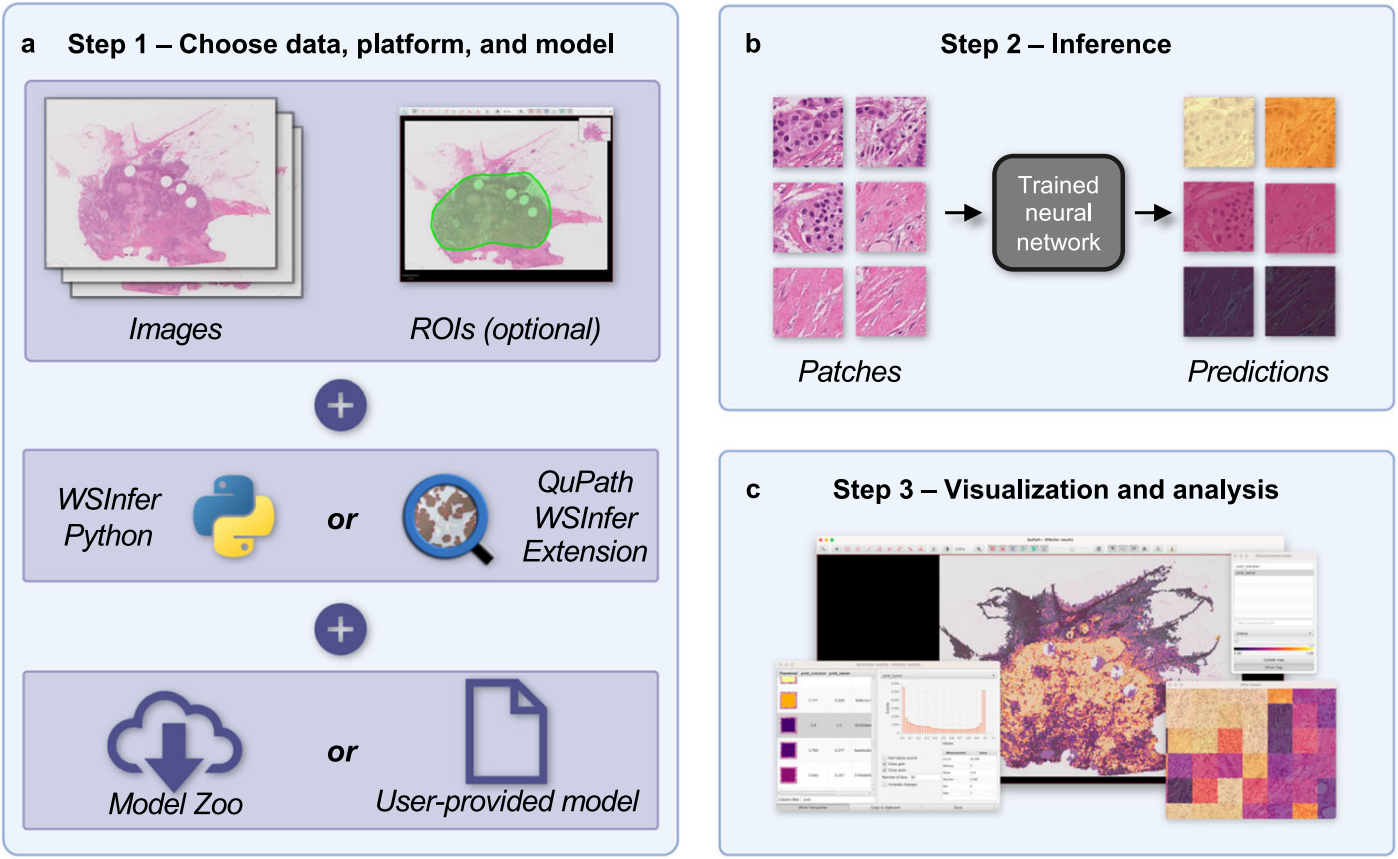
WSInfer workflow. The WSInfer ecosystem streamlines the deployment of trained deep neural networks on whole slide images through three steps. **a** In Step 1, users begin by selecting their WSIs and specifying the platform for model inference along with the choice of a pretrained model. If employing the WSInfer Python Runtime, the dataset is expected to be a directory containing WSI files. Alternatively, when using the WSInfer QuPath extension, the image currently open in QuPath serves as the input. QuPath users also have the option to designate a region of interest for model inference. The pretrained model can be selected from the WSInfer Model Zoo or users can provide their own model in TorchScript format. **b** In Step 2, WSInfer performs a series of processing steps, including the computation of patch coordinates at the patch size and spacing prescribed by the model. Image patches are loaded directly from the WSI and used as input to the patch classification model. The model outputs are aggregated and saved to CSV and GeoJSON files. **c** In Step 3, model outputs can be visualized and analyzed in QuPath or other software. This example shows breast tumor patch classification on a slide from TCGA.

**Table 1:** Models currently available in the WSInfer Model Zoo.

Tissue type	Model outputs	Training dataset	Citation
Breast	Tumor-negative, tumor-positive	TCGA BRCA	^7^
Colorectal	Background, normal colon mucosa, debris, colorectal adenocarcinoma epithelium, adipose, mucus, smooth muscle, cancer-associated stroma, lymphocytes	NCT-CRC-HE-100K	^13,19^
Lung	Lepidic, benign, acinar, micropapillary, mucinous, solid	TCGA LUAD	^25^
Lymph nodes	Metastasis-negative, metastasis-positive	PatchCamelyon	^13,17,26^
PanCancer	Lymphocyte-negative, lymphocyte-positive	23 TCGA cancer types	^8^
Pancreas	Tumor-negative, tumor-positive	TCGA PAAD	^27^
Prostate	Grade 3, grade 4 or 5, benign	TCGA PRAD	^28^

WSInfer provides an open-source, cross-platform, and cross-language ecosystem to make deep learning methods uniquely accessible and intuitive for a wide range of digital pathology stakeholders. The core inference runtime is developed in Python, making it readily accessible for data scientists and deep learning specialists working in digital pathology — for whom Python is typically the programming language of choice. By also providing an extension in the widely adopted QuPath software, we aim to greatly broaden access. We anticipate that making the application of models more streamlined in this way will encourage more pathologists to try the methods on new data. This should, in turn, make it easier to identify strengths and weaknesses, and thereby accelerate the critical feedback loop necessary to develop robust and generalizable algorithms that might improve diagnostic, prognostic, and predictive capabilities.

Several tools exist for deploying trained models on whole slide images, including TIA Toolbox^13^, MONAI^14^, SlideFlow^15^, and PHARAOH^16^. WSInfer complements these by specifically targeting highly optimized, user-friendly support for patch-based WSI inference methods. We expect that these tools may be used together and are keen to promote interoperability. To this end, the WSInfer Model Zoo implements a minimal model configuration specification that accompanies each trained model, with the intention that it may be used by other software beyond the direct WSInfer ecosystem. We host several trained patch classification models in the Zoo, including two models from TIA Toolbox, and intend to incorporate more models in future work. There are other important tasks in digital pathology, including pixel classification, nucleus detection, and slide-level inference. In future work, we plan to include such models. We are actively developing slide-level classification models as well as methods to distribute them in a reusable and reproducible manner.

It is important to note that WSInfer itself supports a variety of patch classification models but is agnostic to a user’s choice of model. It is intended for research use only, and we make no claims regarding the suitability of the models for specific applications. Hence, users assume the responsibility of verifying the suitability of any model for their purposes. Indeed, it is our expectation that promising digital pathology methods will often be found not to perform well on new images; generalization across cohorts, scanners, and laboratories is a hard problem. However, we believe that an important first step to addressing this must be to enable existing models to be properly scrutinized by the research community, to identify what does and does not work. We hope that WSInfer may help further this aim to benefit the wider digital pathology community, and ultimately patients.

One current use case with potential clinical value is the spatial identification of TILs. There is evidence that TILs are prognostic and predictive in a variety of cancers^6,10–12^, and patch classification deep learning models have been developed for the identification of tumor and lymphocyte regions. One can use these models via WSInfer. With the QuPath WSInfer Extension, for example, one may label tumor regions with one of the tumor models in the Zoo, and additionally use a lymphocyte patch classification model in the same region. The spatial maps produced by these models can allow one to visualize the extent of lymphocyte infiltration. In colorectal cancers, this can be done with a single model that classifies tumor and lymphocytes simultaneously (see the “Colorectal” model in Table 1). In the online documentation for the QuPath WSInfer Extension, we include a script that may be used to create these TIL maps (see “Code Availability”).

Another potential use case is screening hematoxylin-and-eosin-stained tissue images. For example, one might screen sentinel lymph node sections for breast cancer metastasis using a model trained on the CAMELYON dataset^13,17,18^. This may assist pathologists in detecting the presence of metastatic cells across entire whole slide images. In addition, WSInfer includes several tumor patch classification models, which may aid pathologists in identifying and measuring tumor regions. WSInfer also includes a model that classifies colorectal tissue patches into multiple phenotypes (e.g., tumor, stroma, lymphocytes, adipose, normal mucosa)^13,19^, and these outputs can be visualized as a map of tissue phenotypes. These may aid pathologists form impressions of tissue and identify regions that require further attention.

The use cases of WSInfer are primarily driven by the models available, and as such we anticipate that the range of applications will expand over time with the addition of future models. A topic of current work is the incorporation of models predicting microsatellite instability status^20^ and genomic aberrations^21^. This may expand the potential predictive and prognostic uses of WSInfer. In the future, we plan to incorporate specimen-level deep learning models, which render a prediction for an entire WSI, as well as models for pixel classification and nucleus detection.

## Methods

WSInfer comprises three main components: (1) the WSInfer inference runtime, (2) the QuPath WSInfer extension, and (3) the WSInfer Model Zoo. Together these provide tools designed to meet the needs of a diverse range of users, including pathologists, computational researchers, and data scientists.

### Inference runtime

The WSInfer inference runtime deploys trained patch classification deep learning models on whole slide images and is available as a command-line tool and Python package. It requires three inputs from the user: a directory of whole slide images, a trained patch classification model, and a directory in which to write results. One may use a model from the Zoo or provide a local trained model Each WSI undergoes a series of processing steps motivated by ref. ^22^. First, patches are extracted from tissue regions at a uniform size and physical spacing. Next, the patches are run through the forward pass of the deep learning model. The runtime saves model outputs in comma-separated values and GeoJSON files. These output files can be used for downstream analyses or visualized using other software, including QuPath.

We timed WSInfer in two environments: one with an enterprise-grade Quadro RTX 8000 GPU on RedHat Linux and the other with a consumer RTX 2080 Ti GPU on Windows Subsystem for Linux (Windows 11 and Debian 12). In both cases, we used the breast tumor classification model “breast-tumor-resnet34.tcga-brca” from the WSInfer Model Zoo and WSIs from The Cancer Genome Atlas. The model uses 350 × 350-pixel patches at 0.25 micrometers per pixel. In the enterprise environment, analysis of 1061 WSIs took 6 h and 46 min, or *23* *s per WSI* (median tissue area = 173 mm^2^). In the second environment, we applied the same model to 30 WSIs, a subset of the initial 1061. The running time was 14 min and 17 s, or *29* *s per WSI* (median tissue area = 179 mm^2^).

### QuPath extension

QuPath is a popular open-source software platform for bioimage analysis^23^. QuPath’s support for visualizing, annotating, and analyzing whole slide images has led to the software being widely adopted within the digital pathology community: to date, it has been downloaded over 400,000 times and cited in over 2400 studies. We therefore developed the QuPath WSInfer Extension as an alternative inference engine to make patch-based classification widely accessible within a familiar, intuitive, and interactive user interface.

The QuPath WSInfer Extension introduces patch-based deep learning support to QuPath the first time, building upon the software’s existing features to provide an end-to-end analysis solution. Users are guided through the steps of selecting a deep learning model and one or more regions of interest for inference. The extension will then proceed to download the model if required, generate tile objects, and run inference (powered by Deep Java Library and PyTorch) at the appropriate resolution and patch size. The user can then visualize the tile classifications and view interactive maps of predicted class probabilities. Furthermore, the tiles can be reused to run inference using additional models, making it possible to integrate information across models. In this way, for example, TILs may be identified using a tumor model and a lymphocyte model. Finally, because the user has access to all QuPath’s other features (e.g. for tile merging, cell segmentation, data export), WSInfer can be integrated into sophisticated QuPath analysis pipelines, which are run either interactively or through automated scripts. We provide an example script on the documentation website that applies a tumor model and lymphocyte model to produce a spatial map of tumor and TILs, and it is possible that such maps may, in the future, assist pathologists in estimating likelihood of treatment response.

The extension can use a GPU if one is installed and if CUDA software is installed (please see “Code Availability” for a link to documentation including installation instructions). A GPU provides fast processing but may not be available for many users. We measured the running time of the QuPath extension using the breast tumor classification model “breast-tumor-resnet34.tcga-brca” with CPU and GPU. Running time was *6* *min 37* *s* on a 100 mm^2^ region of interest using an Intel© Core™ i5-12600K processor in Windows 11 with QuPath v0.4.4 and extension v0.2.1. The same region took *40* *s* using an NVIDIA RTX 2080 Ti GPU in the same environment.

### Model zoo

We curated a collection of trained pathology models for broad, unencumbered reuse and have hosted this Zoo on Hugging Face Hub. Each model repository contains a model card^24^, pretrained weights in TorchScript format, and a configuration JSON file. The model card is a markdown file with human-readable metadata including the purpose of the model, its architecture, description of training data, how to apply it to new data, intended uses, and relevant citations. TorchScript is a serialization format that contains weights and a graph of the forward pass of the model, and it allows the use of the model without a Python dependency. To add a model to the Zoo, one creates a new model repository on Hugging Face Hub and uploads a model card, TorchScript file of the model, and configuration JSON file. One may optionally upload other files as well. Crucially, the user owns the model repository and can license and manage the contents independently. The registry of models in the Zoo is maintained as a JSON file in a dedicated public repository on Hugging Face Hub. After publishing a model on Hugging Face Hub, one may submit a pull request to this repository adding the model location to the registry.

We have also developed a client utility to enhance the interoperability of the Zoo with other software. The client is available as a Python package or command-line tool and primarily lists and downloads models from the Zoo. The client can also validate Model Zoo repositories and model configuration JSON files, functionalities we hope will ease the use of WSInfer.

### Reporting summary

Further information on research design is available in the Nature Research Reporting Summary linked to this article.

### Supplementary information

REPORTING SUMMARY

## Data Availability

The results published here are in whole or part based upon data generated by the TCGA Research Network: https://www.cancer.gov/tcga. Whole slide image files used in runtime benchmarks may be downloaded from https://portal.gdc.cancer.gov/projects/TCGA-BRCA. The whole slide image file shown in Fig. 1 is hosted at https://portal.gdc.cancer.gov/files/d46167af-6c29-49c7-95cf-3a801181aca4. The Model Zoo Registry is available at https://huggingface.co/datasets/kaczmarj/wsinfer-model-zoo-json, and all currently available models can be found at https://huggingface.co/kaczmarj.

## Code availability

The WSInfer Python Runtime is developed for Python 3.8+ and is available under the Apache 2.0 license (https://github.com/SBU-BMI/wsinfer). We provide a user guide for the command-line tool at https://wsinfer.readthedocs.io/en/latest/user_guide.html. The QuPath WSInfer extension is also open-source software, written in Java (https://github.com/qupath/qupath-extension-wsinfer). Please refer to https://qupath.readthedocs.io/en/stable/docs/deep/wsinfer.html for documentation of the QuPath WSInfer extension, including a step-by-step guide, guidance for GPU support, and example scripts demonstrating the use of the extension in analysis pipelines.
